# Identifying barriers and facilitators to the inclusion of older adults living in UK care homes in research: a scoping review

**DOI:** 10.1186/s12877-023-04126-3

**Published:** 2023-07-20

**Authors:** Brittany Nocivelli, Victoria Shepherd, Kerenza Hood, Carolyn Wallace, Fiona Wood

**Affiliations:** 1 Division of Population Medicine, PRIME Centre Wales, School of Medicine, Cardiff University, Cardiff, Wales; 2 Centre for Trials Research, School of Medicine, Cardiff University, Cardiff, Wales; 3 School of Care Sciences, University of South Wales, Newport, Wales

**Keywords:** Care home, Residential home, Nursing home, Older adults, Barriers, Facilitators, Research, Inclusion, Participation, Scoping review

## Abstract

**Background:**

With an ageing population, older adults will have more complex health and social care needs and many of these older adults will be living in care homes. Despite the growth in care home populations, care home residents are often excluded from research that could potentially benefit their care. The purpose of this scoping review is to explore resident-related barriers and facilitators to including older people living in UK care homes in research and to identify potential approaches to modify such barriers.

**Method:**

The 6-stage scoping review methodology framework proposed by Arksey and O’Malley guided this review. Five electronic databases (MedLine, PsychINFO, Scopus, Web of Science, CINAHL) and grey literature were searched. Identified articles went through two levels of screening, and those deemed relevant were collated, summarised and reported using a thematic analysis approach.

**Results:**

90 reports were eligible for inclusion and were synthesised into 7 themes and related subthemes: (1) research design; (2) understanding and beliefs about research (resident and care home staff); (3) communication; (4) relationships; (5) eligibility criteria (resident and care home); (6) preference-based decisions; and (7) care home staff and environment. Given the complex interplay of the factors identified, both direct and indirect factors were included.

**Conclusions:**

A number of recurring barriers and facilitators to the inclusion of care home residents in research are reported. However, isolating resident-related barriers was complex as both direct and indirect factors must be considered as influential. Understanding the barriers and facilitators to inclusion will enable these factors to be addressed and increase the evidence-base for care provided to older people living in care homes.

## Introduction

It has been estimated that by 2037, adults over the age of 65 will account for 24% of the UK population [1]. There are already an estimated 490,326 care home residents in the UK [2–4]. As a result of the ageing population, many more older adults may require the level of support provided by care homes [5].

Far less research is conducted in care homes compared to other healthcare settings, despite twice as many people living in care homes as there are hospital beds in the UK [6, 7]. Additionally, it has been reported that care home staff generally have less access to research training and support [7]. Staff would likely benefit from the development of interventions to support the creation of environments where opportunities for resident participation in research is able to take place and can be integrated into care [7]. Research priorities in care homes have been identified in previous research, including the need for better individualised and person-centred care [8].

Older adults, who often experience the most disease and require the most complex care needs, are generally underrepresented in research [9]. This results in research evidence that may not be generalisable to those who may require it the most [10, 11]. Although the prevalence of chronic health problems increases with age [12], older adults are often excluded from research due to both explicit and implicit restrictions, for example age limits or decisional capacity abilities [13, 14]. If research findings are to effectively inform practice, study participants should reflect the population to which the research is being applied [15]. Furthermore, there is a lack of research which has identified appropriate research methodology and strategies for recruiting older adult populations [15]. Underrepresentation and exclusion of older adults in research is apparent in facilities dedicated to the care of older adults, such as care homes [6].

The exclusion of care home residents in research has been suggested to be partly due to practical difficulties and ethical concerns about including this ‘vulnerable’ group in research [16]. However, all people have the right to be included in research regardless of their place of residence or cognitive abilities. According to the Alzheimer’s Society, 80% of older adults living in care home are estimated to have either dementia or severe memory problems (17). A high number of care home residents therefore lack the capacity to consent to research and are less likely to be included in research as a result. Where care home residents are included, it is often through proxy decision-makers, who may have little knowledge of what their views and attitudes may be or find the process too difficult, thereby limiting residents’ opportunities to express their own wishes [17, 18]. Proxy decision-makers, often termed personal consultees or personal legal representatives, refer to people who are engaged in caring for the participant (not professionally or for payment) or are interested in their welfare and are prepared to be consulted [19].

A previous systematic review, published in 2018, identified a number of challenges to conducting research in care homes [20]. The challenges were categorised into eight main themes: facility/owner factors; resident factors; staff caregiver factors; family caregiver factors; investigator factors, ethical/legal factors; methodological factors; and budgetary factors. The reasons for the exclusion of care home residents are multi-factorial, including structural inequalities from less research infrastructure and research capacity, a reduced research-orientated culture, and individual resident-related factors, such as cognitive impairment [20]. Reference to UK based studies or resident-related challenges were also primarily nested within a larger study, which limits the findings due to international differences in care homes and residents and thus the transferability of studies. The available international literature reporting challenges to conducting research in care homes is limited due to the fact that care homes, care provision and care home residents differ considerably between different countries [21, 22]. Further research is needed to explore these challenges with a focus on care home residents themselves. This will enable greater opportunities for research inclusion for residents, subsequently allowing them to have their voices heard, and receive quality, evidence-based care in the future [23].

To better understand why older adults living in UK care homes are often excluded, and therefore underrepresented, in research, this scoping review aimed to:

- identify resident-related barriers and facilitators to including older people living in UK care homes in research.
- identify potential approaches to appropriately modify identified barriers and facilitators.

The term ‘care home’ is used throughout this paper to refer to any long-term care facilities that older adults live in full time. This includes care homes, residential homes, and nursing homes.

## Methods

### Protocol and Registration

The protocol for this scoping review followed the scoping review protocol framework by Peters et al. (2022; [24]) and can be found at: https://osf.io/fdy78.

### Design

This review follows the scoping review methodology framework proposed by Arksey and O’Malley (2005; [25]) with recommendations from updated versions of the framework by Levac et al. (2010; [26]) and the Joanna Briggs Institute [24, 27] taken into consideration when relevant. According to the methodological framework there are six different stages to consider when undertaking a scoping review: identifying the research question; identifying relevant studies; selecting studies; charting the data; collating, summarising, and reporting the results; and consultation. Whilst the consultation stage is suggested as optional by Arksey and O’Malley, it was included in this study in order to strengthen the findings and their relevance.

The broad nature of a scoping review, as discussed by Munn et al. [28] was deemed the best fit for this review from which some basic concepts in the research area, as well as key sources, concepts, gaps, and the amount and nature of available literature need to be identified. Guidelines from the Preferred Reporting Items for Systematic Reviews and Meta-Analyses, Scoping Review extension (PRISMA-ScR; [29, 30]) were also followed in this review.

#### Stage 1: Identifying the Research Question.

The research question driving this scoping review was: “What are the resident-related barriers and facilitators to including older people living in UK care homes in research?”

#### Stage 2: Identifying Relevant Articles.

For the purpose of consistency, the term ‘articles’ will be used throughout to refer to included materials (published papers, websites, protocols, blogs).

Eligibility Criteria. The identification of relevant articles followed the Population, Concept, Context (PCC) framework (see Table 1.), as recommended by the JBI [24, 27]. Articles were included in the review if they: (1) included care home residents, residents’ family members, care home staff, or researchers; (2) mentioned barriers or facilitators to inclusion, or suggestions/advice for modifying barriers or facilitators; and (3) took place in UK care home settings. In line with the broad nature of the review, no limits were placed on study design. Conference proceedings, protocols and systematic and literature reviews were excluded; however, the reference lists of review articles were searched to ensure that no key articles were missed. Only English language articles were included in this review considering the language abilities of the researchers, as well as time and cost constraints. Searches of all sources were confined to articles published between January 2005 and the date the searches were conducted (March 2022). This time limit ensured that the literature reviewed was relevant to the Mental Capacity Act (2005; [20]) before which the process for including people who lacked capacity to consent was not formalised. The Mental Capacity Act governs how incapacitated adults can be involved in research and provides for another person to be consulted for advice before an individual lacking capacity is included in the research [31]. The geographic context for the search was limited to the UK as different countries have different types of residential care for older adults. Additionally, different countries have different legal frameworks for research involving adults lacking capacity to consent.

**Table 1:** Proposed inclusion criteria for scoping review relevant to PCC framework

	Inclusion Criteria
**Participants/Population**	Care home residents Care home residents’ relatives Care home staff Researchers
**Concept**	Barriers and/or facilitators to inclusion Resident-related factors
**Context**	UK care homes (residential homes, nursing homes, long-term care facilities)
**Type of Source**	Journal articles and other reports, both peer and non-peer reviewed Date of publication between 2005 and review commencement (March 2022) Published in English

Information Sources and Search Strategy. Electronic database searches of: Medline, Web of Science, Scopus, CINAHL and PsychINFO, were conducted by BN on 23-25th March 2022. A combination of terminologies, separated by key concepts, were tailored to each database with the help of a subject specific librarian. See Table 2. for search strategy.

Additionally, grey literature was investigated through unpublished literature (EthOS), whole site searches of relevant organisations (ENRICH, AlzheimersUK, British Society of Gerontology) as well as existing networks. Whole site searches were conducted using a Google search tool recommended by a consulted subject specialist librarian (‘search term:website’).

**Table 2:** Proposed search terminologies to be input into each database, separated by key concept

	Key Concepts		Search terms
	Care homes (titles and abstracts)	OR	“care home*”, “nursing home*”, “residential home*”, “long-term care facilit*”
**AND**	Research (titles)	OR	“research*”, “study*”, “trial*”, “investig*, “explor*”, “observ*”
**AND**	Participation (titles and abstracts)	OR	“research subject*”, “research particip*”, “particip* research”, “recruit*”, “involv*”
**AND**	Barriers and facilitators (titles and abstracts)	OR	“barrier*”, “challeng*”, “factor*”, “facilitat*”, “perception*”, “perceive*”, “view*”, “attitude*”, “experience*”

#### Stage 3: Selecting Articles.

One author (BN) performed the screening after having piloted implementing the eligibility criteria alongside another author (VS) with a random selection of articles. In screening level one, the title and abstract were reviewed for eligibility. During screening level two, the full article was reviewed against the eligibility criteria and advice was sought from another author (VS) for any articles where inclusion was unclear. Any disagreement about inclusion between BN and VS was referred to another author (FW) for discussion and resolution.

#### Stage 4: Charting the Data.

Data were extracted from the included articles according to the following fields: author(s) and year; source type; purpose; population; concept (barriers and facilitators); context; relevant author suggestions/advice for modification; and any other relevant comments.

The data charting form was taken from scoping review resources developed by the JBI (https://jbi-global-wiki.refined.site/space/MANUAL/4687579) and modified as relevant, per instruction of the JBI. Data charting for all included articles was completed independently by BN, with feedback provided by FW and VS.

After further familiarisation with the articles, barriers and facilitators were extracted and the number of articles that discussed each factor was recorded.

#### Stage 5: Collating, Summarising, and Reporting the Results.

Following identification of the barriers and facilitators to inclusion of care home residents in research, factors were placed into categories based upon the system level to which they were related (i.e., staff-related, resident-related, care home-related, research-related). Although aiming to identify resident-related barriers and facilitators only, due to the complex interactions with other system-level factors other intersecting and influential indirect factors were included. Each of the barriers and facilitators identified therefore fell into either direct or indirect categories, all with the potential to impact the inclusion of UK care home residents in research. Following familiarisation with the barriers and facilitators identified in the included articles, as is usual with scoping review methodology [29], the themes and sub-themes were iteratively developed through discussion with the team.

#### Stage 6: Consultation.

An online meeting was held in January 2023 with stakeholders to discuss the initial draft of the scoping review. The meeting included five participants, three of whom were Patient and Public Involvement (PPI) group members identified through Health and Care Research Wales. Perspectives shared by the stakeholder patient and public involvement members included those of care home staff, care home resident relative, and researcher.

A brief presentation of the scoping review was sent to members a week in advance with instructions to consider contributing input in the meeting based around their own expertise and perspectives. The aim of this consultation meeting was to clarify and/or validate our preliminary findings. The same presentation was shared in the meeting and members shared and discussed their own thoughts and perspectives, based on their own experiences, of the information presented.

The PPI group were consulted earlier on in the project during the initial stages of identifying barriers and facilitators to the inclusion of older adults living in UK care homes in research and so were familiar with the project and able to contribute valuable views.

## Results

**Fig. 1:**
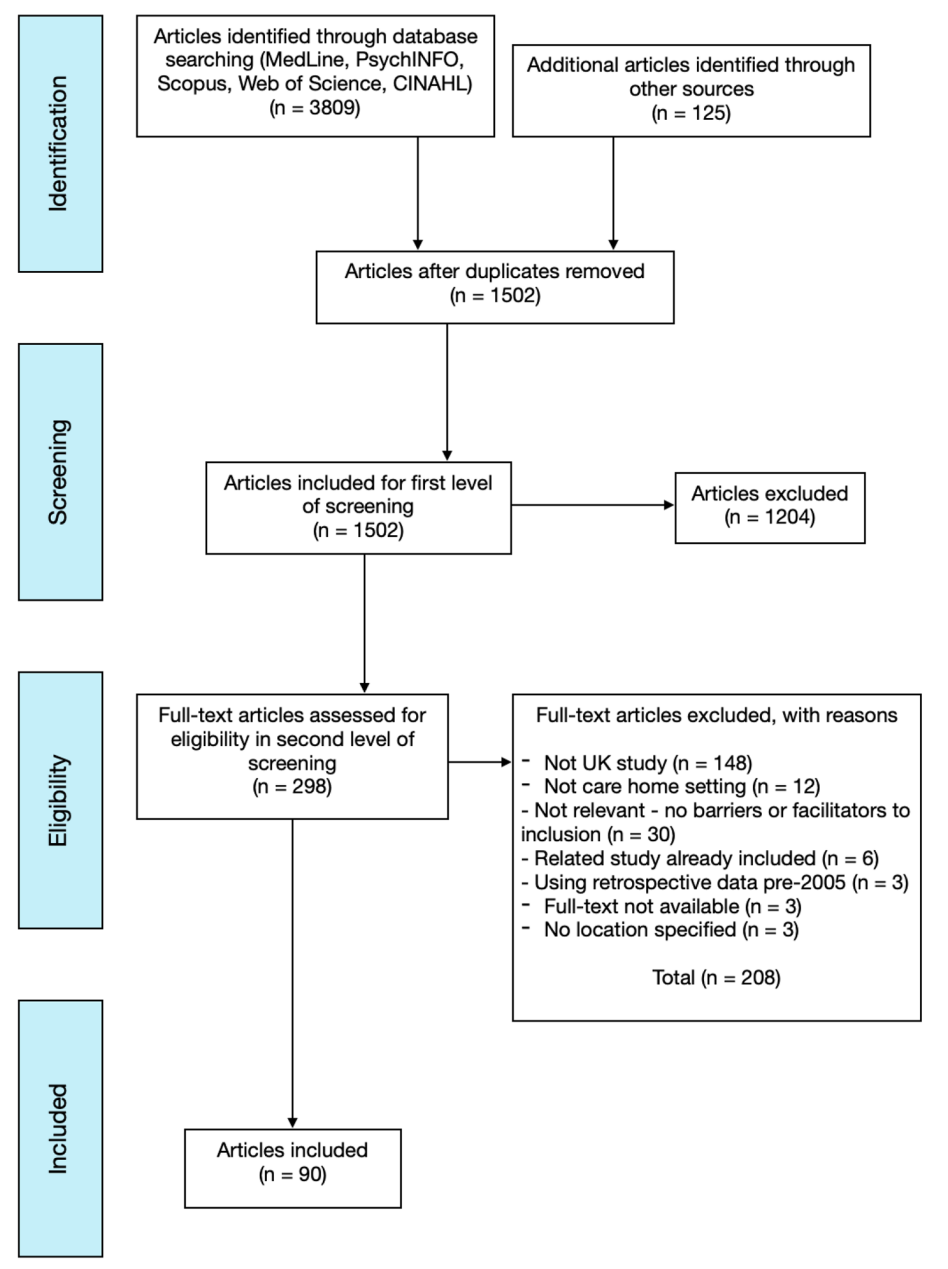
PRISMA-ScR flow chart of article selection

A total of 3809 articles were identified from the database searches and a further 125 from grey literature and other sources (see Fig. 1. for PRISMA-ScR flow chart). Following deduplication of articles, 1525 articles remained. All articles were uploaded to a reference management system, Endnote, where data management and both screening levels were completed against the eligibility criteria. After the screening of titles and abstracts during screening level 1, using the predefined eligibility criteria, a total of 1204 articles were excluded, resulting in 313 articles. Following the second level of screening, 223 were excluded based on full-text review, resulting in 90 articles for data extraction.

### Article characteristics

The general characteristics of the articles included in this scoping review are reported in Table 1. 3809 journal articles and 125 articles from the grey literature search were initially retrieved. Of the 90 articles included, 84 reported potential barriers and 75 reported potential facilitators of inclusion of UK care home residents in research (see Table 3). Of the included articles, 30 also included advice or suggestions for improving the inclusion of care home residents in research (see Table 4).

### Barriers and facilitators to the inclusion of UK care home residents in research

Alongside resident-related factors that directly affected the inclusion of care home residents, a number of indirect factors were identified which were viewed as important and influential and so warranted inclusion. Factors directly affecting inclusion refers to factors which are solely related to and impact the resident, such as cognitive impairment, whereas indirect factors to inclusion refer to impactful factors that residents have no control over and may even be unaware of, such as gatekeeping.

The complex barriers and facilitators to the inclusion of UK care home residents in research were synthesised into seven thematic categories: (1) research design; (2) understanding and beliefs about research (resident and care home staff); (3) communication; (4) relationships; (5) eligibility criteria (resident and care home); (6) preference-based decisions; and (7) care home staff and environment. See Table 5.

### Research Design

A number of research design issues were discussed in the included articles, which posed barriers and facilitators to the inclusion of care home residents in research.

The use of existing networks during recruitment was a common approach and resulted in being an indirect facilitator to the inclusion of care home residents in research [32–51]. However, the sole use of existing networks, including ‘research ready’ care homes for example, may also present an indirect barrier for the inclusion of UK care home residents in research [32, 35, 46, 50], as the approach excludes those care homes that are not within those networks.

The piloting of the recruitment process was mentioned in two of the included articles and poses a potential indirect facilitator to inclusion [33, 51]. Piloting was considered helpful in terms of identifying challenges which can be addressed prior to recruitment. Researcher flexibility, including tailoring research methods and/or requirements to specific care home settings and/or residents was discussed in a number of included reports [47, 48, 17, 52], as was the importance of researcher experience in care home settings [53].

The research design choice of relying on care home staff to determine study eligibility was commonly reported by the included articles, posing a potential barrier to the inclusion of care home residents in research through issues of recruitment bias [37, 38, 40, 43, 54, 67]. Further, the burden, on care home residents and staff, of the chosen methods of data collection, including monitoring periods were discussed in included articles [39, 17, 52, 68, 69], as were designs which require significant time and environmental requirements [53, 70, 71], such as private space, all of which present potential barriers to the inclusion of care home residents in research.

### Understanding and beliefs about research (resident and care home staff)

#### Resident

A number of the included articles discussed barriers around residents’ general lack of interest in participating in research, as well as initial interest and then disengagement [35, 36, 46, 17, 55, 59, 70, 72, 73]. Resident understanding about what research is, what is required of them, and other related concerns also posed a potential barrier for inclusion [74–76]. Highlighting to residents the potential benefits of research was the most common facilitator discussed in the included articles [17, 52, 70, 77–79], followed by residents’ altruism [52, 74].

#### Care Home Staff

A lack of understanding by care home staff and negative beliefs about research, including underlying research motives were discussed in a number of included articles [34, 39, 45, 53, 59, 71, 80, 81]. Ensuring accurate understanding about the nature of the research being conducted, and staff having positive beliefs about the research was reported in a number of included articles and offered a potential indirect facilitator to resident inclusion [47, 75, 81].

### Communication

The approach to presenting research information to potential participants was discussed in some of the included articles, posing both a potential barrier and facilitator to the inclusion of care home residents in research [59, 74]. Communicating information to residents in an accessible, tailored manner was considered to be a direct facilitator to resident inclusion [45, 55, 59, 68, 70, 74, 82]. Providing clear and honest information from the start, as well as facilitating positive, clear and consistent communication with all stakeholders were factors also considered to be helpful [47–49, 51, 52, 65, 67, 70, 71, 74–76, 78, 80, 83]. One included article discussed the importance of effective communication ensuring true understanding [75]. Difficulties in communication, including those caused by cognitive impairment and loss of verbal skills were reported as direct barriers for inclusion in research for care home residents [74, 77]. Fluctuations in resident capacity and in resident mood also posed challenges to participation in research [17, 55, 72, 73].

Poor communication between care home staff, researchers, and relatives posed another potential indirect barrier to inclusion [17, 80], as did poor communication between the research team and staff [33, 48, 49, 71, 74, 77, 81].

### Relationships

The importance of building rapport between the research team, residents, care home staff and relatives was discussed in many included articles. The importance of researchers spending time at care homes before study commencement was particularly commonly discussed and is a potential facilitator to inclusion [34, 43, 67, 71, 74, 82, 84, 85]. The benefits of developing positive relationships with gatekeepers, such as care home managers, were discussed also [65, 69].

The use of a collaborative working style between the research team, residents, staff, and relatives proposed a potential facilitator to the inclusion of care home residents in research [45, 50, 52, 55, 59, 61, 65, 68, 70, 78, 82, 83, 86–89]. Providing personalised feedback and a feeling of inclusivity for care home staff and residents was also mentioned as a positive experience and may indirectly facilitate resident inclusion in research [47, 49].

### Eligibility criteria (resident and care home)

#### Eligibility of Residents

Strict resident eligibility criteria were the most common direct resident-related barriers to inclusion, with exclusion often based on age limits [32–39, 54–58, 68, 90–101] and comorbidity (e.g., learning disability, terminal illness, cognitive impairment) being the most common [38, 40, 41, 54, 55, 58–60, 68, 74, 75, 77, 90, 92, 93, 96–109]. The exclusion of participants who lacked the capacity to consent to participation, with no option of utilising a personal consultee, were reported [34, 37, 38, 42–44, 61–63, 68, 72, 93, 98, 103, 109] as well as those who did not have an adequate ability to communicate, understand, or engage in conversation [37, 44, 45, 58, 60, 102, 103, 107]. The requirement of a clinical diagnosis of dementia (as opposed to a likely diagnosis) was a potential barrier in a number of included articles [36, 41, 56, 57, 98, 101–103, 107, 109–111], as was the requirement to understand and communicate in English [34, 41, 43, 45, 55, 58, 60, 62, 74, 75, 95, 102, 103, 107, 109]. The requirement of a study partner posed a potential barrier was discussed in two articles [52, 109].

The allowance of another person being able to consent to participation on behalf of a resident who lacks the capacity to consent, i.e., a personal consultee, was the most frequently mentioned potential facilitator to inclusion in the included articles [32, 40, 41, 45, 53, 57, 58, 59, 64, 68, 74, 75, 77, 79, 82–86, 88, 90–92, 94, 95, 97, 100, 101, 105–108, 111–116]. Additionally, utilising minimal eligibility criteria was also found to be a potential facilitator to the inclusion of care home residents in research [32, 42, 44, 50, 52, 64, 79, 84, 88, 91, 104, 106, 110, 113, 114, 117].

#### Eligibility of Care Homes

The presence of strict care home eligibility criteria proposed an indirect resident-related barrier to inclusion for UK care home residents. Most commonly reported were the need to meet criteria for the location and type of care home [33, 34, 41, 42, 44, 46, 56, 76, 86, 109] and [33, 34, 37, 38, 42, 44, 46, 56, 76, 77, 112, 113], respectively. The size of care homes was another common eligibility criteria [33, 37, 41, 45, 57, 84, 90], as were the rating/quality of care homes, as awarded by organisations such as the Care Quality Commission [33, 36, 37, 45, 47, 66, 74, 75, 110]. Care homes who were requiring special support from their local authorities were also reported to be excluded from some research [110, 111].

### Preference-Based Decisions

Residents’ expressions of perceptions of disempowerment, including lack of autonomy, confidence, apathy and having worries about research participation were discussed in a number of included articles and posed barriers relating to participation in research [45, 55, 59, 61, 64, 74, 111]. Further, a lack of awareness about research participation opportunities and being overlooked with regards to participation posed potential barriers to inclusion [52, 74, 118]. Providing residents with the opportunity to participate in research, by directly asking them, is a potentially empowering facilitator to inclusion which was discussed in one article [74].

Relatives’ unwillingness to take part, or in cases where a personal consultee option was available, refused to consent or make a decision regarding resident participation, presented a barrier to inclusion [17, 39, 55, 83, 86, 90, 119], as did the impact of what article authors referred to as “gatekeeping” and “overprotective relatives” [17, 54, 65, 69, 70, 74, 77, 87, 91, 112, 117].

The impact of external influences was discussed in included articles and were potential indirect barriers to research inclusion. The impact of research ethics committees was discussed in one article [53], as was the impact of legal frameworks [119].

### Care Home Staff and Environment

Factors relating to the care home, including the care home staff and the care home environment creates both direct and indirect barriers and facilitators to the inclusion of care home residents in research.

Providing and communicating the benefits and incentives of research participation to care home staff was mentioned in a number of included articles and may provide an indirect facilitator to research inclusion [47, 49, 51, 52, 71, 106]. Care home staff interest, support, and engagement in research were reported to provide an indirect facilitator to research inclusion [39, 47, 51, 52, 55, 68, 70, 71, 76, 81, 101, 108, 116], as did care home manager interest specifically [103, 114]. A number of included articles also discussed the benefits of providing staff training and opportunities for knowledge development as part of the research process [47, 51, 70, 72, 76].

The impact of research on care home staff was the most common indirect resident-related barrier to inclusion, with time pressure felt by care home staff and workload factors most commonly discussed [43, 47–49, 53, 59, 65, 71, 73, 75, 76, 78, 80], followed by high staff turnover [39, 49, 52, 53, 65, 70, 71, 78, 81, 83]. Staff lack of interest, engagement and negative attitudes towards research, were the next most frequently discussed [39, 45, 47, 53, 55, 59, 75, 77, 81]. A lack of confidence in facilitating research was discussed in two included articles [59, 84]. Perceived lack of support from the care home manager [34, 74, 75, 78, 81] and the culture within care homes [52, 54] were also discussed in included articles. Conversely, manager support for the study was reported as an indirect facilitator [74, 77, 78, 81, 102, 108].

Limitations of the care home environment, including a lack of private space in which to consent residents and collect data, and disruption of daily routines caused by research, posed a barrier to resident inclusion [34, 54, 55, 59, 65, 67, 73, 74, 77, 78]. However, in a number of included articles, it was shown that the care home environment can be used to facilitate research participation, such as positive use of spaces that were chosen by residents, for example residents’ own bedrooms, to conduct research which facilitates privacy [17, 61, 65, 74, 104]. However, residents’ ability to have their own private room is not always available in all care homes.

Furthermore, the culture of care homes, specifically care homes with a culture of inclusiveness, was reported as a facilitator to the inclusion of residents in research [45].

### Consultation Stage

When presenting our early synthesis to our PPI partners, we received comments about our choice of vocabulary, much of which reflected terms used by the authors of the literature included in the review. For example, the use of the word ‘overprotective’ in relation to relatives was disliked by one member, stating that it felt harsh and unfair.

Suggestions of additional visualisations of the results were made, such as the inclusion of a graphic showing the weighting of barriers and facilitators depending on how many times each came up in the included literature. The inclusion of a table stating which barriers could be tackled most easily compared to those more difficult to tackle was discussed also.

Further discussion related to one member’s own experiences of working in different types of care homes. For example, for researchers to consider that care home staff may have different time and workload demands dependent upon whether they are working in a residential or nursing home.

Overall, the discussion supported our preliminary findings, including the importance of care home staff as a factor. PPI members expressed their interest in taking part in the review process and shared their views on the importance of the topic throughout. One member shared their own experiences of visiting a relative living in a care home and the apparent issues of recruitment and pressures of high workload. This member also shared the view that staff often do not have English as a first language, making them more cautious towards research, and that it may be a lower priority for them as it contributes towards their already high workload. The facilitatory benefits of researchers spending time in care homes prior to study commencement was discussed and strongly agreed with by the group members. A suggestion for future research surrounding the topic of how to facilitate conversation between researchers and care home staff about research and its benefits was made by one member.

Changes made in light of the consultation stage included:

- The clarification of our definition of ‘care homes’ as homes which care is provided for older adults and not other types of care homes which might provide care for younger adults with disabilities.
- Adding more information to clarify that terms which may be less favourable, such as ‘overprotective’ have been used as these were terms used in the literature.
- Including the suggestion of exploring the topic of how to facilitate conversation between researchers and care home staff in future research.

## Discussion

This scoping review set out to understand why older adults living in UK care homes are often excluded, and therefore underrepresented, in care home research with the aim of identifying resident-related barriers and facilitators to their inclusion and identify potential interventions to appropriately modify identified barriers and facilitators. The barriers and facilitators identified in the existing literature have been collated, synthesised, and reported in this review.

The majority of included articles were research articles conducted in care home facilities, although there were also a number of commentary articles from researchers about the processes of conducting research in care homes. Frequently reported barriers and facilitators to the inclusion of care home residents in research were grouped into seven thematic categories: (1) research design; (2) understanding and beliefs about research (resident and care home staff); (3) communication; (4) relationships; (5) eligibility criteria (resident and care home); (6) preference-based decisions; and (7) care home staff and environment. Approaches or solutions we suggest in light of these findings are presented in Table 5.

### Barriers

Barriers to the inclusion of care home residents in research were mainly related to factors outside of the residents’ control, such as research methods and the communication and relationships between research systems and care systems.

The use of existing networks during recruitment, whilst beneficial when used alongside other methods of recruitment, poses a barrier when used as the sole method of recruitment. For example, the use of ‘research ready’ care homes results in the exclusion of the majority of care homes in the UK that we know are not registered as ‘research ready’ or actively engaging with research.

Strict eligibility criteria for participation, both for residents and for care homes, were identified in a majority of the included articles. Whilst necessary for any study to provide eligibility criteria in order to focus their population of interest, strict criteria relating to characteristics of care home residents, such as age, prevents the inclusion of residents that could otherwise provide a representative sample of the targeted population. The potential impact of excluding representative participants based on characteristics which may be unrelated to the research aim, or interfere with the research findings, may be unfavourable in relating findings to practice. Further, strict eligibility criteria for care homes, such as size, rating/quality and type limit research opportunities from even reaching care home residents who represent a population who reside in the variety of care homes available in the UK. This is in line with discussion by Patino and Ferreira (2018; [120]) regarding the impact of inclusion and exclusion criteria on the external validity of a study.

The lack of an opportunity for a relative or personal consultee to consent on behalf of residents who lacked capacity to consent to their own participation presented a barrier to inclusion. It is likely that including extra stages to obtain informed consent from those lacking capacity can be both time-consuming for researchers and present additional costs. This finding is in line with research which suggests that care home research can be challenging to conduct due to practical difficulties and ethical concerns [121]. Other practical difficulties and ethical concerns were identified from the review relating to the impact of external factors such as legal frameworks and research ethics committees. These findings are in line with a recent review of barriers and facilitators by Ritchie et al. (2023 [122]), which discusses data privacy regulations as a barrier to recruitment causing care home staff to involuntarily act as ‘gatekeepers’. Ritchie and colleagues suggest that by establishing residents’ and representatives’ preparedness to be approached at the point of care home admission, this barrier could be removed. Further, relatives’ unwillingness to take part in care home research or their refusal to consent on behalf of, or make a decision on, their relatives’ participation posed a barrier to resident inclusion. It may be possible that by establishing stakeholders’ preparedness at the point of care home admission, as suggested by Ritchie and colleagues, this barrier can be overcome.

More barriers than facilitators were identified in this scoping review relating to the theme of preference-based decisions. Residents’ lack of awareness of opportunities to participate in research were shared by a number of included articles and present an important barrier suggesting that current recruitment strategies are ineffective. Whilst research generally aims to investigate and discover ways in which we can improve quality of life of a target population, there is a paucity of research aiming to understand how care home residents feel about and understand the purpose and benefits of research, thus in some cases impacting their willingness to contribute or participate. Expressions of disempowerment by residents, where they questioned their abilities to contribute in a useful way to research, was apparent in the included articles alongside apparent lack of autonomy, confidence, apathy and worries about research participation. According to Self Determination Theory (SDT; Deci & Ryan, 1985 [123], 1991 [124]), perceived autonomy can result in feelings of empowerment and improve motivation to carry out tasks which are felt to be a product of one’s own choice. Improving perceived autonomy of older adults living in care homes could be beneficial in this research area. Informing and educating older adults living in care homes about research, and how they can be involved, may be a useful step towards increasing opportunities for inclusion.

### Facilitators

Not surprisingly, this review has identified that a number of facilitators to care home resident inclusion in research correspond to identified barriers. For example, poor communication between researchers and residents, relatives and care home staff resulted in more barriers, whereas clear, consistent, and positive communication between individuals and organisations were a facilitator to resident inclusion. Further, researchers providing personalised feedback and a feeling of inclusivity for staff and residents was reported in the included literature as a positive experience for stakeholders. Ritchie et al. (2023 [122]) also identified challenges relating to communication between the research team and care home staff outside of the care home setting. Furthermore, difficulties in communication experienced by residents, which may pose a barrier to inclusion, can be rectified through the presentation of research information in an accessible and tailored manner, thus facilitating inclusion. Researchers are responsible for modifying most factors which present as barriers to the inclusion of care home residents in research. Researcher flexibility and experience working with care homes and residents is of great importance in tackling challenges.

Within the theme of relationships, a number of other facilitators were identified. The use of a collaborative working style between all stakeholders was discussed as beneficial in a number of articles as beneficial as were the benefits of developing positive relationships with gatekeepers, such as care home managers. Building rapport with stakeholders, for example by researchers spending time in care homes before study commencement, was a facilitator identified in a number of included studies. These findings are aligned with reports of beneficial research outcomes of collaborative working styles in other health care settings [125].

Within the care home staff and environment theme, capitalising on the unique care home environment such as private rooms and communal social spaces, can facilitate resident inclusion, as shown in some of the included articles. In addition, the high workload and time pressures faced by staff, identified in the included articles, may be addressed by manager support of the research study making researchers aware of the most suitable times to carry out research related tasks. Investing in staff development through training may facilitate positive staff engagement in research, which was identified as a facilitator to the inclusion of care home residents in research. This finding is in line with Gordon et al. (2022 [126]), who suggest that investing in the development of the care home workforce can help to make staff feel more valued and give them the recognition they deserve to match the importance of their work.

Further, by removing additional research pressures, care home staff may be more willing to facilitate resident recruitment. This flexibility relates to suggestions from other included articles, stating that patience, flexibility and need for understanding complexities of care home environments are key researcher qualities needed for successful recruitment and data collection.

### Strengths and Limitations

In accordance with scoping review methodology, we did not include an assessment of the methodological quality of included articles. However, the aim of this review was to identify underlying concepts in the research area, as well as key sources and the nature of available literature [29], for which a scoping review was the most appropriate approach [24]. Whilst a large amount of literature was identified, we identified a number of common themes which allows confidence in our application of the broad yet rigorous scoping review methodology.

Although a comprehensive search was carried out, with a focused but inclusive search strategy, it is possible that all published articles in this area were not identified.

A strength of this review is the inclusion of both direct and indirect barriers and facilitators which were identified during data extraction and are thought to have a great impact on older adults’ inclusion in research. Other strengths include that data were included from a wide range of study types and stakeholders’ experiences, enabling the findings to be drawn from these wider perspectives rather than those of individuals studies or groups. A further strength of this scoping review was the inclusion of the consultation stage of Arksey and O’Malley’s methodology framework which allowed the exploration and clarifying of our preliminary findings using additional expertise and perspectives of stakeholders.

### Future Research and practical implications

This scoping review provides new insights on the barriers and facilitators to UK care home residents’ research participation presented in the existing literature. Many of the barriers have the potential to be modified, thus improving recruitment and inclusion. It may be of interest for future research to investigate barriers and facilitators for different types of care home or for residents with differing characteristics (e.g., those with capacity to consent and those without). Furthermore, future research may also consider the different barriers to the inclusion of care home residents in research depending on the type of research methodology (e.g., randomised controlled trials vs. survey).

Apparent from the findings of this review was a lack of literature reporting the views of relevant stakeholders (i.e., residents, relatives, staff, and researchers) about the opportunities for older adults living in care home to get involved in research.

Future research may also consider focusing on the development of a simpler process of involving people with capacity to consent in research, with a specific focus on care home residents. This would need to include individuals living with dementia who represent the majority of older adults living in care homes.

Furthermore, future research to explore how residents’ wishes and feelings about research participation, and the quality of understanding about research by this population may be useful in improving recruitment practice.

Finally, attempts to address the identified barriers to resident inclusion can be made using the solutions identified in this review. Tools have recently been developed which aim to help researchers to design trials that are more inclusive of particular underserved populations (e.g., the INCLUDE Ethnicity Framework [127], and the INCLUDE Impaired Capacity to Consent Framework [128]) but have not yet been applied to trials being conducted in care homes. If these are successful, researchers may expect their results to be more generalisable to this underrepresented population who may benefit the most.

## Conclusions

Care home residents remain an under-served group in research, which results in less evidence about how to best care for this group than those receiving care in other settings. This scoping review identified a number of complex, interacting barriers and facilitators to the inclusion of older adults living in UK care homes in research.

The findings have enabled a better understanding of common barriers and facilitators to the inclusion of care home residents in research, as well as presenting potential ways these factors can be modified to improve research within the field.

Further research is required in order to explore the interaction between the direct and indirect barriers and facilitators to UK care home resident inclusion in research and identify interventions that target the modifiable barriers and facilitators to improve inclusion.

**Table 3:** General characteristics of included articles

Author(s)	Year	Article type	Purpose/Title	Location	Setting	Participant/Perspective	Barriers	Facilitators	Advice included
NIHR (ENRICH)	2015	Interview blog	Overcoming the challenges of recruiting care homes to research	UK-wide	N/A	Researcher	✔	✔	✔
NIHR (ENRICH)	2015	Interview blog	Talk to the people who know - consulting widely before starting care home research	UK-wide	N/A	Researcher		✔	✔
Aguirre et al.	2012	Intervention study	Cognitive simulation therapy (CST) for people with dementia - who benefits most?	London, Essex, and Bedfordshire, UK	Care homes and community settings	113 care home residents	✔		
Airlie, Forster and Birch	2022	Randomised Controlled Trial	An investigation into the optimal wear time criteria necessary to reliably estimate physical activity and sedentary behaviour from ActiGraph wGT3X + accelerometer data in older care home residents	West Yorkshire, UK	Care homes	94 care home residents	✔	✔	
Amador et al.	2014	Observational Study	Emergency ambulance service involvement with residential care homes in the support of older people with dementia: An observational study	East of England, UK	Care homes	133 care home residents	✔	✔	
Aspray et al.	2006	Survey study	Low bone mineral density measurements in care home residents—a treatable cause of fractures	Newcastle upon Tyne, UK	Care homes	392 care home residents	✔	✔	
Ballard et al.	2018	Randomised Controlled Trial	Impact of person-centred care training and person-centred activities on quality of life, agitation, and antipsychotic use in people with dementia living in nursing homes: A cluster-randomised controlled trial	South London, North London, and Buckinghamshire, UK	Care homes	757 care home residents	✔	✔	
Barber et al.	2009	Prospective study	Care homes’ use of medicines study: Prevalence, causes and potential harm of medication errors in care homes for older people	West Yorkshire, Cambridgeshire, and central London, UK	Care homes	256 care home residents		✔	
Bartlett, Milne and Croucher	2019	Reflective paper	Strategies to improve recruitment of people with dementia to research studies	UK-wide	N/A	Researchers	✔	✔	✔
Butler et al.	2020	Randomised Controlled Trial	Effect of Probiotic Use on Antibiotic Administration among Care Home Residents: A Randomized Clinical Trial	UK	Care homes	310 care home residents	✔	✔	
Carter et al.	2008	Observational Study	Chronic kidney disease prevalence in a UK residential care home population	East Kent, UK	Residential homes	250 care home residents	✔	✔	
Churcher et al.	2017	Pilot intervention study	An adapted mindfulness intervention for people with dementia in care homes: Feasibility pilot study	UK	Care homes	31 care home residents	✔		
Clarke et al.	2019	Interview study	A qualitative interview study comparing and contrasting resident and staff perspectives of engaging in meaningful activity in a UK care home	South London, UK	Care homes	9 care home residents, 11 care home staff members	✔	✔	
Close et al.	2013	Interview study	“It’s Somebody else’s responsibility” - perceptions of general practitioners, heart failure nurses, care home staff, and residents towards heart failure diagnosis and management for older people in long-term care: a qualitative interview study	Northeast England, UK	Residential and care homes	17 care home residents, 8 care home staff	✔	✔	
Costa, Ockelford and Hargreaves	2018	Mixed methods qualitative study	The effects of listening to preferred music on symptoms of depression and anxiety amongst elders in residential care: A qualitative, mixed methods study	London, UK	Care homes	113 residents	✔	✔	
Cunneen et al.	2011	Observational study	An investigation of food provision and consumption in a care home setting	East of Scotland, UK	Care homes	25 care home residents	✔	✔	
Davies et al.	2014	Reflective paper	Enabling research in care homes: An evaluation of a national network of research ready care homes	UK-wide	N/A	Researcher	✔	✔	✔
Donnelly et al.	2017	Qualitative study	Burden of a Remote Trial in a Nursing Home Setting: Qualitative Study	Dublin, Ireland, UK	Care homes	11 care home residents, 10 care staff members	✔	✔	
Ellmers	2011	Thesis	A qualitative study of sleep and the night-time in care homes for older people	Guilford, UK	Care homes	38 care home residents, 39 care home staff members	✔		
Ellwood et al.	2018	Reflective paper	Recruiting care homes to a randomised controlled trial	UK-wide	N/A	Researcher	✔	✔	
Evans et al.	2011	Reflective paper	Evaluating services in partnership with older people: Exploring the role of ‘community researchers’	UK-wide	N/A	Researcher	✔	✔	
Ferguson	2020	Thesis	Supporting older people living in care homes: a qualitative network approach	Scottish Central Belt, UK	Care homes	36 care home residents	✔	✔	
Fleetwood-Smith, Tischler and Robson	2021	Reflective paper	Using creative, sensory and embodied research methods when working with people with dementia: a method story	UK-wide	N/A	Researcher	✔	✔	✔
Forster et al.	2021	Randomised Controlled Trial	An intervention to increase physical activity in care home residents: results of a cluster-randomised, controlled feasibility trial (the REACH trial)	Yorkshire, UK	Care homes	152 care home residents	✔	✔	✔
Fossey et al.	2020	Qualitative study	“We should see her like part of the team”: An investigation into care home staff’s experiences of being part of an RCT of a complex psychosocial intervention	London, Oxfordshire, and Buckinghamshire, UK	Care homes	41 care home staff members	✔	✔	
Gallagher et al.	2017	Action Research	Realising dignity in care home practice: An action research project	South of England, UK	Care homes	Care home staff		✔	✔
Gillespie et al.	2015	Prospective cohort study	Antibiotic prescribing and associated diarrhoea: a prospective cohort study of care home residents	South Wales, UK	Care homes	279 care home residents	✔	✔	✔
Gine-Garriga et al.	2020	Interview study	Mission (im)possible: Engaging care homes, staff and residents in research studies	Glasgow, UK	Care homes	2 care home staff members	✔	✔	
Godfrey et al.	2012	Qualitative study	An exploration of the hydration care of older people: a qualitative study	Southwest England, UK	Care homes	5 care home residents	✔		
Goodman et al.	2013	Qualitative study	Preferences and priorities for ongoing and end-of-life care: A qualitative study of older people with dementia resident in care homes	East of England, UK	Care homes	18 care home residents	✔	✔	
Goodman et al.	2011	Reflective paper	Culture, consent, costs and care homes: Enabling older people with dementia to participate in research	UK-wide	N/A	Researcher	✔	✔	✔
Gordon et al.	2014	Cohort study	Health status of UK care home residents: a cohort study	Nottingham, UK	Care homes	227 care home residents	✔	✔	
Graham et al.	2020	Randomised Controlled Trial	A posture and mobility training package for care home staff: results of a cluster randomised controlled feasibility trial (the PATCH trial)	Yorkshire, UK	Care homes	146 care home residents	✔	✔	
Griffiths et al.	2019	Trial process evaluation	Barriers and facilitators to implementing dementia care mapping in care homes: results from the DCM TM EPIC trial process evaluation	West Yorkshire, Oxford, and London	Care homes	726 care home residents	✔	✔	✔
Hall et al.	2019	Qualitative study	Moving beyond ‘safety’ versus ‘autonomy’: a qualitative exploration of the ethics of using monitoring technologies in long-term dementia care	Northern England, UK	Care homes	3 care home residents, 24 care home staff members, 9 relatives	✔	✔	
Hall and Beatty	2014	Interview study	Assessing spiritual well-being in residents of nursing homes for older people using the FACIT-Sp-12: A cognitive interviewing study	London, UK	Care homes	17 care home residents	✔		
Hall et al.	2013	Qualitative study	‘It makes me feel that I’m still relevant’: A qualitative study of the views of nursing home residents on dignity therapy and taking part in a phase II randomised controlled trial of a palliative care psychotherapy	London, UK	Care homes	49 care home residents	✔		
Hall et al.	2011	Qualitative study	Implementing a quality improvement programme in palliative care in care homes: a qualitative study	London, UK	Care homes	11 care home residents, 26 care home staff members, 7 relatives	✔	✔	
Hall, Longhurst and Higginson	2009	Reflective paper	Challenges to conducting research with older people living in nursing homes	Southeast London, UK	Care homes	18 care home residents	✔	✔	✔
P. Higgins	2013	Reflective paper	Involving people with dementia in research	UK-wide	N/A	Researcher	✔	✔	✔
Horne et al.	2018	Reflective paper	Improving trial recruitment in care homes: the Falls IN Care Homes (FINCH) experience	UK-wide	N/A	Researcher	✔	✔	
Hsu et al.	2015	Randomised controlled feasibility study	Individual music therapy for managing neuropsychiatric symptoms for people with dementia and their carers: a cluster randomised controlled feasibility study	UK	Care homes	17 care home residents, 10 care home staff members	✔	✔	
Jain et al.	2021	Qualitative study	Dog-assisted interventions in care homes: A qualitative exploration of the nature, meaning and impact of interactions for older people	Southeast of England, UK	Care homes	54 care home residents	✔	✔	
Jenkins et al.	2016	Reflective paper	Overcoming challenges of conducting research in nursing homes	UK-wide	N/A	Researcher	✔	✔	✔
LaFrenais	2015	Reflective paper NIHR blog	Understanding Care Home Research	UK-wide	N/A	Researcher	✔	✔	✔
Law	2016	Thesis	Research in care homes: issues of participation and citizenship	Scotland, UK	Care homes	Researcher	✔	✔	✔
Law et al.	2021	Survey study	Motivating and constraining factors for research participation in Scottish care homes	Scotland, UK	Care homes	Care home staff	✔	✔	
Law and Ashworth	2022	Interview study	Facilitators and Barriers to Research Participation in Care Homes: Thematic Analysis of Interviews with Researchers, Staff, Residents and Residents’ Families	Scotland, UK	Care homes	12 care home residents, 15 care home staff members, 6 relatives, 8 researchers	✔	✔	
Lee and Bartlett	2021	Ethnographic study	Material Citizenship: An ethnographic study exploring object-person relations in the context of people with dementia in care homes	Southern England, UK	Residential home	15 care home residents, 16 care home staff members, 8 relatives		✔	
Livingston et al.	2012	Intervention study	Improving the end-of-life for people with dementia living in a care home: an intervention study	London, UK	Care homes	Care home residents, care home staff members, and relatives		✔	
Luff et al.	2015	Reflective paper	A guide to research with care homes (2015)	UK-wide	N/A	Researchers	✔	✔	✔
Maidment et al.	2018	Intervention study	Medication review plus person-centred care: A feasibility study of a pharmacy-health psychology dual intervention to improve care for people living with dementia	West Midlands, UK	Care homes	108 care home residents	✔	✔	✔
Maluf	2017	Thesis	The social lives of older men living in care homes and the implications for their wellbeing	UK-wide	Care homes	Care home residents, care home staff members, relatives	✔	✔	
Moore et al.	2017	Intervention study	Implementing the compassion intervention, a model for integrated care for people with advanced dementia towards the end of life in nursing homes: a naturalistic feasibility study	Northern London, UK	Care homes	9 care home residents	✔		
NIHR	2019	Blog post/interview	Helen’s Story	UK-wide	N/A	Researcher	✔		
O’Neill et al.	2022	Interview study	‘Waiting and Wanting’: older peoples’ initial experiences of adapting to life in a care home: a grounded theory study	UK-wide	Care homes	17 care home residents	✔		✔
Orellana et al.	2019	Qualitative study using interviews and observations	Older care home residents’ and their relatives’ knowledge, understanding and views of shift handovers: an exploratory, focused-ethnographic qualitative study using interviews and observations	Southeast England, UK	Care homes	10 care home residents, 5 care home managers, 6 relatives	✔		
Orrell et al.	2007	Randomised Controlled Trial	A cluster randomised controlled trial to reduce the unmet needs of people with dementia living in residential care	London, North Wales, and Manchester, UK	Care homes	238 care home residents	✔		
Paddock et al.	2019	Qualitative case study using interviews and observations	Care Home Life and Identify: A Qualitative Case Study	Greater Manchester, UK	Care homes	9 care home residents, 4 relatives, 5 care home staff members	✔	✔	✔
Parsons et al.	2015	Feasibility study	Development and Application of Medication Appropriateness Indicators for Persons with Advanced Dementia: A Feasibility Study	Northern Ireland, UK	Care homes	15 care home residents	✔	✔	
Patchwood, et al.	2020	Qualitative study using interviews and observations	Six-month reviews for stroke survivors: A study of the modified Greater Manchester Stroke Assessment Tool with care home residents	Northwest of England, UK	Care homes	71 care home residents	✔	✔	
Perfect et al.	2019	Reflective paper	Collecting self-report research data with people with dementia within care home clinical trials: Benefits, challenges and best practice	UK-wide	Care homes	Researcher	✔		✔
Powell et al.	2017	Pilot parallel Randomised Controlled Trial	Pilot parallel randomised controlled trial of protective socks against usual care to reduce skin tears in high risk people: ‘STOPCUTS’	Exeter, Exmouth/Sidmouth, and Mid Devon, UK	Care homes	54 care home residents	✔	✔	
Rajkumar et al.	2016	Factorial Cluster Randomised Controlled Trial	Apathy and Its Response to Antipsychotic Review and Nonpharmacological Interventions in People With Dementia Living in Nursing Homes: WHELD, a Factorial Cluster Randomized Controlled Trial	UK-wide	Care homes	273 care home residents	✔	✔	
NIHR	N/A	Interview/Blog	Taking part in research – the care home perspective	UK-wide	N/A	Researcher/Care home manager	✔	✔	✔
Riazi et al.	2012	Qualitative study	Quality of life in the care home: A qualitative study of the perspectives of residents with multiple sclerosis	Within 100 miles of London, UK	Care homes	37 care home residents	✔	✔	
Richardson et al.	2020	Reflective paper	Research with older people in a world with COVID-19: Identification of current and future priorities, challenges and opportunities	UK-wide	N/A	Researcher	✔	✔	✔
Sackley et al.	2015	Cluster Randomised Controlled Trial	An occupational therapy intervention for residents with stroke related disabilities in UK care homes (OTCH): cluster randomised controlled trial	UK-wide	Care homes	1042 care home residents	✔	✔	✔
Sampson et al.	2018	Prospective cohort study	Living and dying with advanced dementia: A prospective cohort study of symptoms, service use and care at the end of life	Greater London, UK	Care homes	70 care home residents	✔	✔	✔
Shamshirsaz	2015	Thesis	Apply QFD methodology to capture ‘unheard’ voices of UK care home residents and translate them into quality measurement targets for future improvement	Peterborough and West London, UK	Care homes	15 care home residents	✔		
NIHR –Shepherd	2020	Blog post	How care homes can support the inclusion of people with impaired capacity	UK-wide	N/A	Researcher		✔	
Shepherd et al.	2015	Reflective paper	Setting up a clinical trial in care homes: challenges encountered and recommendations for future research practice	UK-wide	N/A	Researcher	✔	✔	✔
Shrotri et al.	2021	Prospective cohort study	Vaccine effectiveness of the first dose of ChAdOx1 nCoV-19 and BNT162b2 against SARS-CoV-2 infection in residents of long-term care facilities in England (VIVALDI): a prospective cohort study	England, UK	Long-term care facilities	10,412 care home residents	✔	✔	
Siddiqi et al.	2016	Feasibility cluster Randomised Controlled Trial	The PiTSTOP study: a feasibility cluster randomized trial of delirium prevention in care homes for older people	UK-wide	Care homes	215 care home residents	✔	✔	✔
Simpson et al.	2017	Feasibility study	The challenges and opportunities in researching intimacy and sexuality in care homes accommodating older people: a feasibility study	Northwest England, UK	Care homes	6 care home residents and their partners, 16 care home staff members	✔	✔	
Smith et al.	2019	Reflective paper	Encouraging managers of care homes for older adults to participate in research	UK-wide	N/A	Researcher	✔	✔	
Stow et al.	2018	Cluster randomised feasibility trial	Care home resident and staff perceptions of the acceptability of nutrition intervention trial procedures: a qualitative study embedded within a cluster randomised feasibility trial	UK-wide	Care homes	4 care home residents, 12 care home staff members	✔	✔	
Subramaniam, et al.	2014	Randomised Controlled Trial	Life review and life story books for people with mild to moderate dementia: A randomised controlled trial	North Wales, UK	Care homes	23 care home residents	✔		
Towers et al.	2019	Cross-sectional study	A cross-sectional study exploring the relationship between regulator quality ratings and care home residents’ quality of life in England	Southeast England, UK	Care homes	293 care home residents	✔	✔	
Tzouvara et al.	2016	Reflective paper	Lessons learned from recruiting nursing homes to a quantitative cross-sectional pilot study	UK-wide	N/A	Researcher	✔	✔	
Underwood et al.	2013	Randomised Controlled Trial	Exercise for depression in care home residents: a randomised controlled trial with cost-effectiveness analysis (OPERA)	Northeast London, Coventry, and Warwickshire, UK	Care homes	891 care home residents	✔	✔	
Usman et al.	2019	Prospective cohort study	Measuring health-related quality of life of care home residents: comparison of self-report with staff proxy responses	East Midlands, England, UK	Care homes	117 care home resident and staff matched pairs	✔	✔	
Watkins et al.	2017	Qualitative interview study	Exploring residents’ experiences of mealtimes in care homes: A qualitative interview study	Southwest England, UK	Care homes	11 care home residents	✔	✔	✔
Wenborn et al.	2013	Cluster Randomised Controlled Trial	Providing activity for people with dementia in care homes: A cluster randomised controlled trial	London, UK	Care homes	210 care home residents	✔	✔	✔
Whelan et al.	2013	Reflective paper	Impact of the demand for ‘proxy assent’ on recruitment to a randomised controlled trial of vaccination testing in care homes	UK-wide	N/A	Researcher	✔		
Windle et al.	2018	Mixed-methods longitudinal investigation	The impact of a visual arts program on quality of life, communication, and well-being of people living with dementia: A mixed-methods longitudinal investigation	Northeast England, UK	Care homes	48 care home residents	✔	✔	
Wood et al.	2013	Qualitative study	Consent, including advanced consent, of older adults to research in care homes: a qualitative study of stakeholders’ views in South Wales	South Wales, UK	Care homes	14 care home residents, 14 relatives, 10 GPs, care home staff	✔	✔	
Wylie et al.	2017	Pilot randomised controlled trial	Podiatry intervention versus usual care to prevent falls in care homes: pilot randomised controlled trial (the PIRFECT study)	East of Scotland, UK	Care homes	43 care home residents	✔	✔	✔
Zamir et al.	2018	Implementation study	Video-calls to reduce loneliness and social isolation within care environments for older people: an implementation study using collaborative action research	Devon and Cornwall, UK	Care homes	8 care home residents	✔	✔	
Zermansky et al.	2007	Reflective paper	Striving to recruit: the difficulties of conducting clinical research on elderly care home residents	UK-wide	N/A	Researcher	✔	✔	✔

**Table 4:** identified barriers and facilitators to the inclusion of UK care home residents in research

Barriers	Facilitators
Research Design	
The sole use of existing networks, including ‘research ready’ care homes for example [32, 35, 46, 50] Care home staff responsible for choosing who they deemed as eligible to participate [37, 38, 40, 43, 54–67] The research burden of the chosen methods of data collection, including monitoring periods were discussed in included articles [39, 17, 52, 68, 69] Designs which require significant time and environmental requirements such as private space [53, 70, 71]	The use of existing networks during recruitment [32–51] Piloting of the recruitment process [33, 51] Researcher flexibility, including tailoring research methods and/or requirements to specific care home settings and/or residents [47, 48, 17, 52] Researcher experience in care home settings [53]
Understanding and beliefs about research	
Resident - Residents’ general lack of interest in participating in research, as well as initial interest and then disengagement [35, 36, 46, 17, 55, 59, 70, 72, 73] - Resident misunderstanding about what research is, what is required of them, and other related concerns [74–76] Care home staff - Lack of care home staff understanding and negative beliefs about research, including underlying research motives [34, 39, 45, 53, 59, 71, 80, 81]	Resident - Highlighting the potential benefits of research [17, 52, 70, 77–79] - Residents’ altruism [52, 74] Care home staff - Ensuring true understanding about the nature of the research being conducted, and staff having positive beliefs about the research [47, 75, 81]
Communication	
The approach to presenting research information to potential participants [59, 74] Difficulties in communication, including those caused by cognitive impairment and loss of verbal skills [74, 77] Fluctuations in resident capacity and in resident mood [17, 55, 72, 73] Poor communication between care home staff researchers, and relatives [17, 80] Poor communication between the research team and staff [33, 48, 49, 71, 74, 77, 81]	The approach to presenting research information to potential participants [59, 74] The communication of research information to residents in an accessible, tailored manner [45, 55, 59, 68, 70, 74, 82] Providing clear and honest information from the very start, as well as facilitating positive, clear and consistent communication with all stakeholders [47–49, 51, 52, 65, 67, 70, 71, 74–76, 78, 80, 83]
Relationships	
	Researchers spending time at care homes before study commencement [34, 43, 67, 71, 74, 82, 84, 85] The benefits of developing positive relationships with gatekeepers, such as care home managers, were [65, 69] The use of a collaborative working style between the research team, residents, staff, and relatives [45, 50, 52, 55, 59, 61, 65, 68, 70, 78, 82, 83, 86–89] Providing personalised feedback and a feeling of inclusivity for care home staff and residents [47, 49]
Eligibility criteria	
Resident - Age limitations [32–39, 54–58, 68, 90–101] - Comorbidity (e.g., learning disability, terminal illness, cognitive impairment) [39, 41, 42, 56, 57, 60–62, 70, 76, 77, 79, 92, 94, 95, 98–111] - The exclusion of participants who lacked the capacity to consent to participation, with no option of utilising a personal consultee [34, 37, 38, 42–44, 61–63, 68, 72, 93, 98, 103, 109] - Exclusion of those who did not have an adequate ability to communicate, understand, or engage in conversation [37, 44, 45, 58, 60, 102, 103, 107] - The requirement of a clinical diagnosis of dementia [36, 41, 56, 57, 98, 101–103, 107, 109–111] - The requirement of an ability to understand and communicate in English [34, 41, 43, 45, 55, 58, 60, 62, 74, 75, 95, 102, 103, 107, 109] - The requirement of a study partner [52, 109] Care home - Location of care home [32, 33, 40, 41, 43, 45, 54, 74, 84, 107] - Type of care home [32, 33, 36, 37, 41, 43, 45, 54, 74, 75, 110, 111] - Size of care homes [33, 37, 41, 45, 57, 84, 90] - Rating/quality of care homes, as decided by organisations such as the Care Quality Commission [33, 36, 37, 45, 47, 66, 74, 75, 110] - Care homes receiving special support from their local authorities were excluded in some included studies [110, 111]	Resident - The allowance of another person being able to consent to participation on behalf of a resident who lacks the capacity to consent, i.e., a personal consultee [32, 40, 41, 45, 53, 57, 58, 59, 64, 68, 74, 75, 77, 79, 82–86, 88, 90–92, 94, 95, 97, 100, 101, 105–108, 111–116] - Utilising minimal eligibility criteria [32, 42, 44, 50, 52, 64, 79, 84, 88, 91, 104, 106, 110, 113, 114, 117]
Preference-based decisions	
Residents’ expressions of perceptions of disempowerment, including lack of autonomy, confidence, apathy and having worries about research participation [45, 55, 59, 61, 64, 74, 111] A lack of awareness about research participation opportunities, and being overlooked with regards to participation [52, 74, 118] Relatives’ unwillingness to take part, or in cases where personal consultee option was available, refused to consent or make a decision regarding resident participation, [17, 39, 55, 83, 86, 90, 119] The impact of gatekeeping and overprotective relatives [17, 54, 65, 69, 70, 74, 77, 87, 91, 112, 117] The impact of research ethics committees [53] The impact of legal frameworks [119]	Providing residents with the opportunity to participate in research, by directly asking them [74]
Care home staff and environment	
Time pressure felt by care home staff and workload factors [43, 47–49, 53, 59, 65, 71, 73, 75, 76, 78, 80] High staff turnover [39, 49, 52, 53, 65, 70, 71, 78, 81, 83] Staff lack of interest, engagement and negative attitudes towards research, participation, and facilitation [39, 45, 47, 53, 55, 59, 75, 77, 81] A lack of confidence in facilitating research was discussed in two included articles [59, 84] Perceived lack of support from the care home manager [34, 74, 75, 78, 81] The culture within care homes [52, 54] A lack of private space and disruption of daily routines caused by research [34, 54, 55, 59, 65, 67, 73, 74, 77, 78]	Providing and communicating the benefits and incentives of research participation to care home staff [47, 49, 51, 52, 71, 106] Care home staff interest, support, and engagement in research [39, 47, 51, 52, 55, 68, 70, 71, 76, 81, 101, 108, 116] Manager interest in research [103, 114] Providing staff training and opportunities for knowledge development as part of the research process [47, 51, 70, 72, 76] Manager support of the research study [74, 77, 78, 81, 102, 108] Positive use of spaces that were chosen by residents, for example residents’ own bedrooms, to conduct research [17, 61, 65, 74, 104] The culture of care homes, specifically care homes with a culture of inclusiveness [45]

**Table 5:** Advice and recommendations taken from included articles for modifying barriers and facilitators

Issues	Proposed solutions
Research Design	Work with stakeholder organisations when designing studies e.g., Care Quality Commission (CQC), local authorities – consider the perspectives of each individual shareholder but also take into account the relationships and hierarchy both within a care home and between it and other organisations and health professionals Embed Public Involvement (PPI) throughout and consider how to support their involvement through taking account of residents’ needs due to cognitive impairment and physical frailty Allow care home staff to play a key role in identifying eligible residents, share information and introduce researchers to residents Consider how the consent arrangements will impact on the study – for example ensuring that residents who lack capacity to consent can participate through the involvement of a consultee or legal representative For each step in recruitment, make extensive plans that build in time, including time to be flexible in the face of unexpected hurdles. Adapt measures or questions to potential participants. Understand that recruitment is a resource intensive process and that it requires a lot of preparatory work. There are many layers of permissions needed to support the recruitment process in care homes Provide training so that staff can better understand how to support decisions about capacity and communication approaches, and ensure person-centred inclusion research processes Understand that the staffing pressure and the unique environment of care homes may impact on research – be patient, flexible, supportive and understand the complexities involved, and minimise additional workload for care home staff and any costs associated with taking part Identify realistic targets with the manager at the start. Take the time to learn about shift patterns and mealtimes – understand that care always comes first, research is not the top priority for staff Researchers should develop their skills in order to support residents with dementia to participate in research Be open, responsive, and sensitive – talk to, and work WITH, care home staff Provide accessible, tailored communication tools in order to have the best chance of supporting residents to understand the research and provide informed consent
Communication	Recognise that staff have an invaluable role in supporting residents to understand information about a study and maximise their ability to provide consent if they want to participate. Staff can act as a bridge for communication and advise researchers on any communication aids, best times to approach etc. Ensure that staff have genuine understanding of the research study, so they share correct information, as well as developing a good relationship with them so that they are happy to help. Consider making them research partners so they feel more included and part of the team Communicate well with the care home so that staff know when researcher is coming so they can plan ahead – provide opportunities for meetings and be transparent Identify realistic targets with the manager at the start. Take the time to learn about shift patterns and mealtimes – understand that care always comes first, research is not a top priority for staff Provide accessible, tailored communication tools in order to have the best chance of getting residents to be fully informed and understand the research – e.g., use of pictorial or print text cards
Relationships	Care home managers can support with recruitment when explaining studies to residents, the early involvement of residents’ families, data collection that takes account of residents’ needs, tailored information and support for care home staff Understand the differences in each care home’s culture. The influence of the culture within a care home may impact on how care home staff engage with the research, define dementia, and interpret their roles as mediators, protectors and gatekeepers Develop good and trusting relationships with staff and demonstrate willingness to work with staff – be a respectful researcher and support staff, be guided by managers and staff, try to allay concerns faces by any of the stakeholders, provide active appreciation through feedback
Eligibility criteria	Avoid intentional and unintentional exclusion of potential participants because of age, multi-morbidity or frailty, or impaired capacity to consent
Preference-based decisions	Utilise legal arrangements that can be put in place if residents want to participate but have no family to act as a consultee/legal representative e.g., ensuring care home staff can act as a consultee/legal representative Provide accessible, tailored communication tools in order to have the best chance of getting residents to be fully informed and understand the research
Care homes	Allow care home staff to play a key role in identifying eligible residents, share information and introduce researchers to residents Staff can act as a bridge for communication Recognise that staff have an invaluable role in supporting residents to understand information about a study and maximise their ability to provide consent if they want to participate Staff can advise researchers on any communication aids, best times to approach etc. Care home managers can support with recruitment when explaining studies to residents, the early involvement of residents’ families, data collection that takes account of residents’ needs, tailored information and support for care home staff Provide training so that staff can better understand how to support decisions about capacity and communication approaches, and person-centred inclusion research processes Become a ‘research ready’ care home

## Data Availability

Supporting data and materials used in this paper can be accessed online through various public databases. The datasets used and/or analyses during the current study are available from the corresponding author on reasonable request.

## Abbreviations

**PPI**: Patient and Public Involvement
**CQC**: Care Quality Commission
**MCA**: Mental Capacity Act
